# Supplementary material for: Growth cone advance requires EB1 as revealed by genomic replacement with a light-sensitive variant
Source: eLife. 2023 Jan 30;12:e84143. doi: 10.7554/eLife.84143 (PMC9917429; doi:10.7554/eLife.84143)

Figure 1C - source data

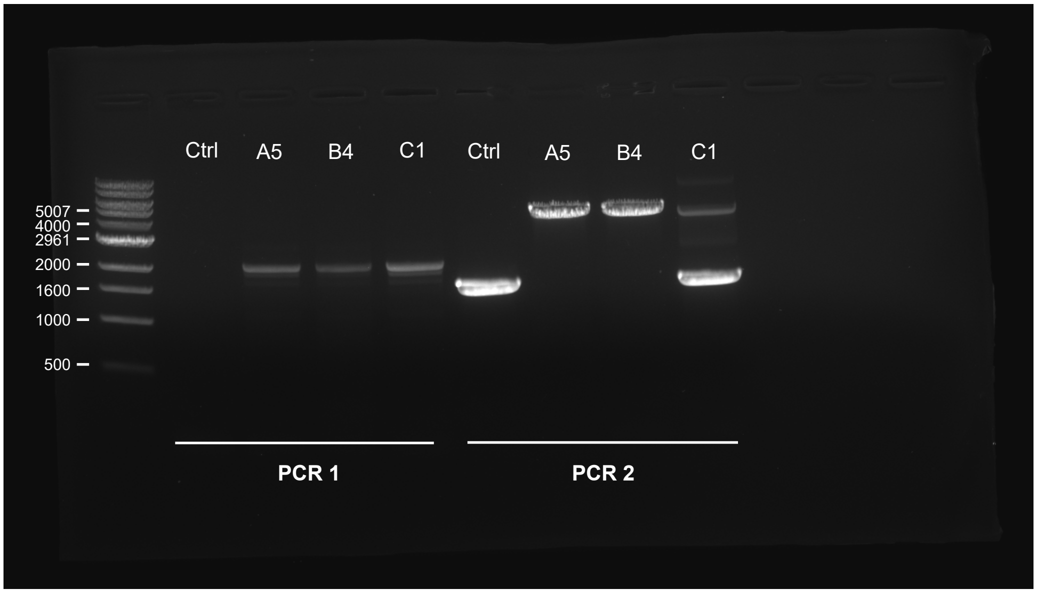

Note: C1 is a not fully edited (heterozygous?) clone that we did not further characterize, and is not included in the main figure.

Figure 1D - source data

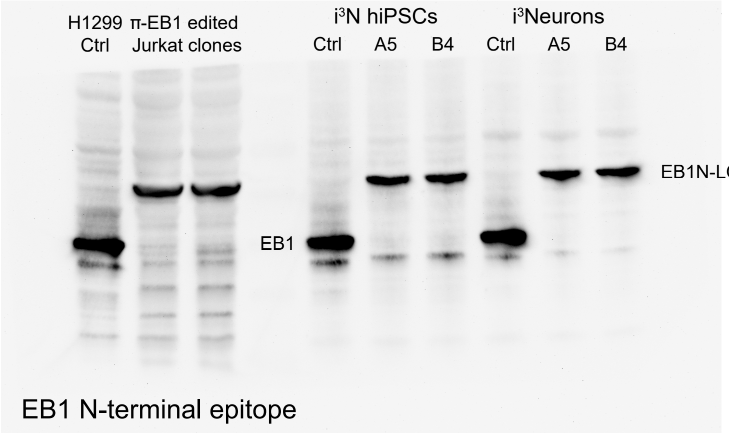

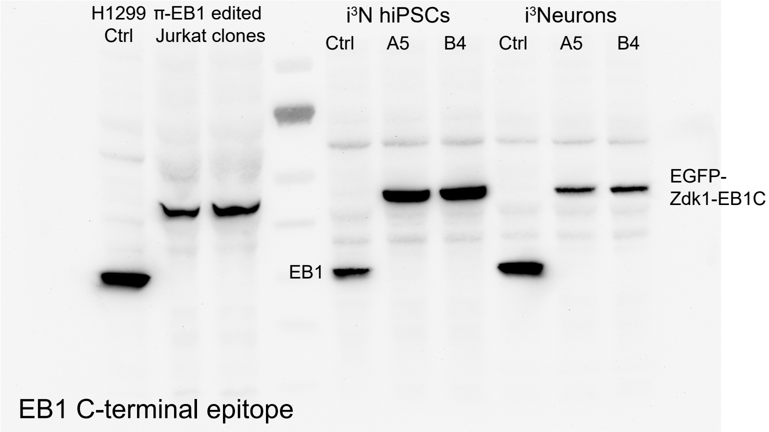

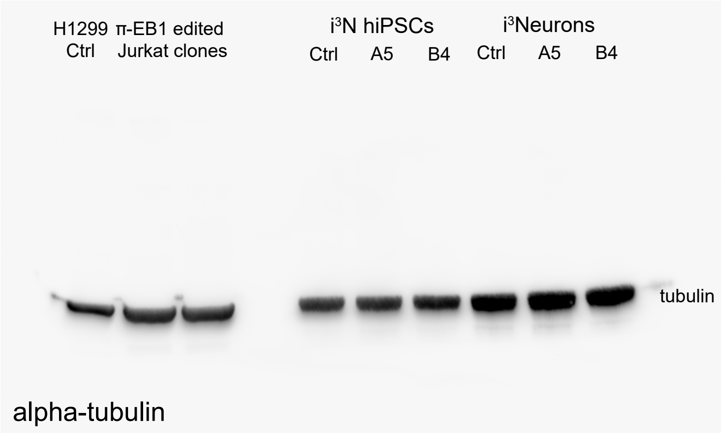

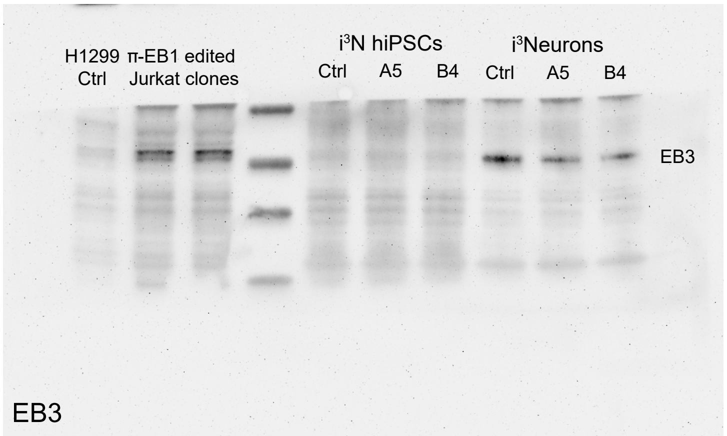

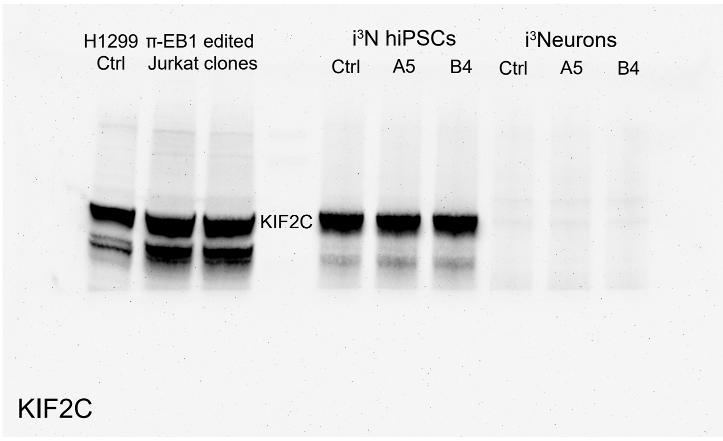

Figure 1H - source data

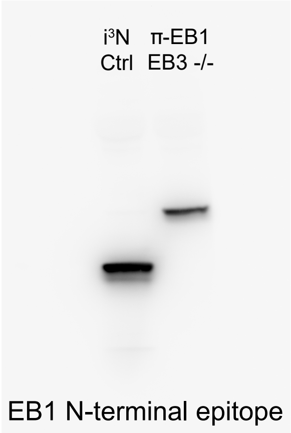

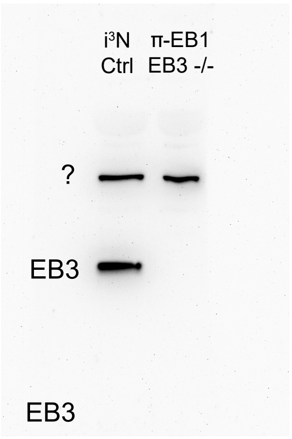

Supplement: Figure 1—source data 1. [file elife-84143-fig1-data1.zip › Figure_1_annotated_source_data.pdf]
