# Supplementary figures and images for: Growth cone advance requires EB1 as revealed by genomic replacement with a light-sensitive variant
Source: eLife. 2023 Jan 30;12:e84143. doi: 10.7554/eLife.84143 (PMC9917429; doi:10.7554/eLife.84143)

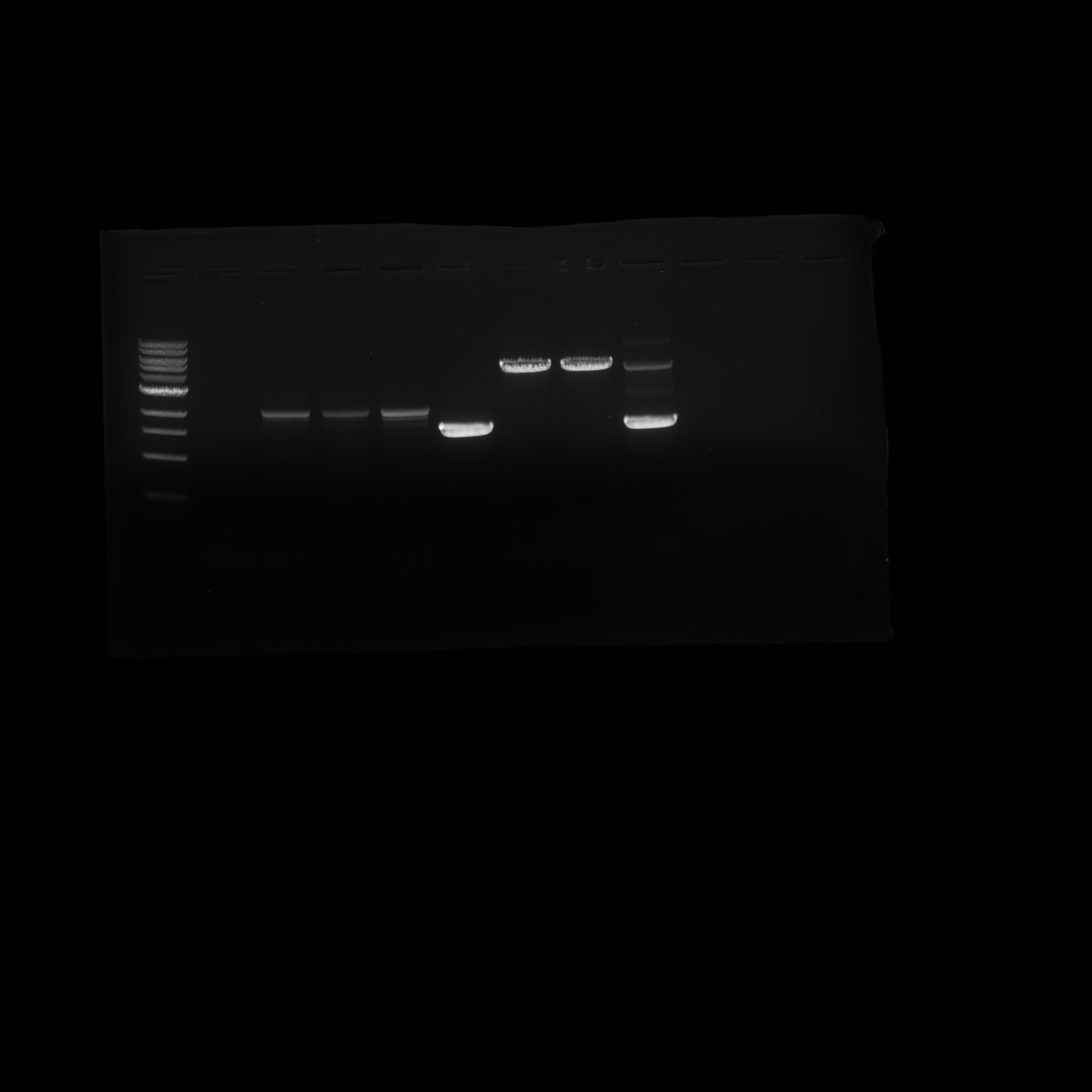

Supplement: Figure 1—source data 1. [file elife-84143-fig1-data1.zip › Fig_1C/04-09-18-PCRiPSpiEB-L3'wt-A5-B4-C1-95+96wt-A5-B4-C1.tif]

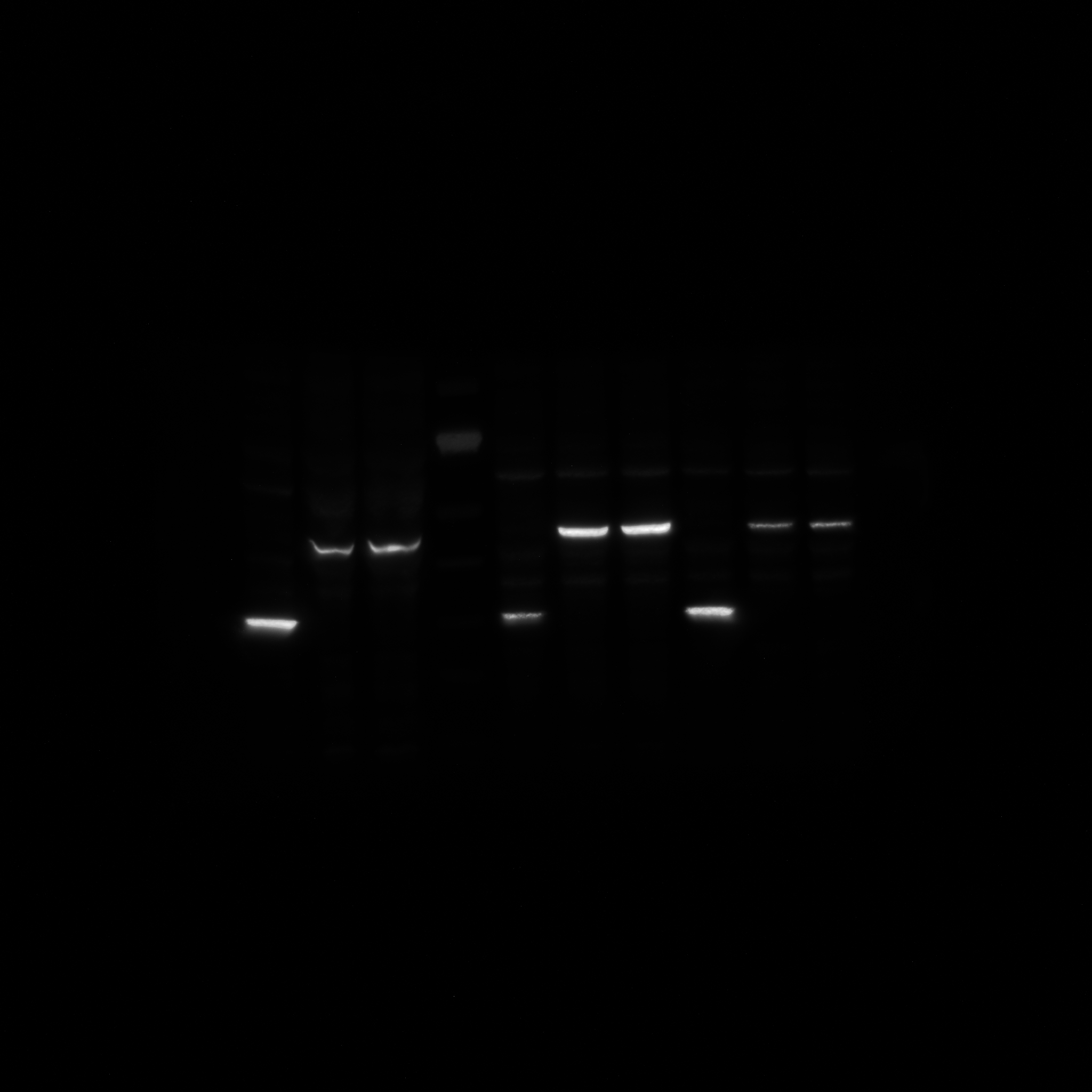

Supplement: Figure 1—source data 1. [file elife-84143-fig1-data1.zip › Fig_1D/EB1C_blot_1min_exposure.tif]

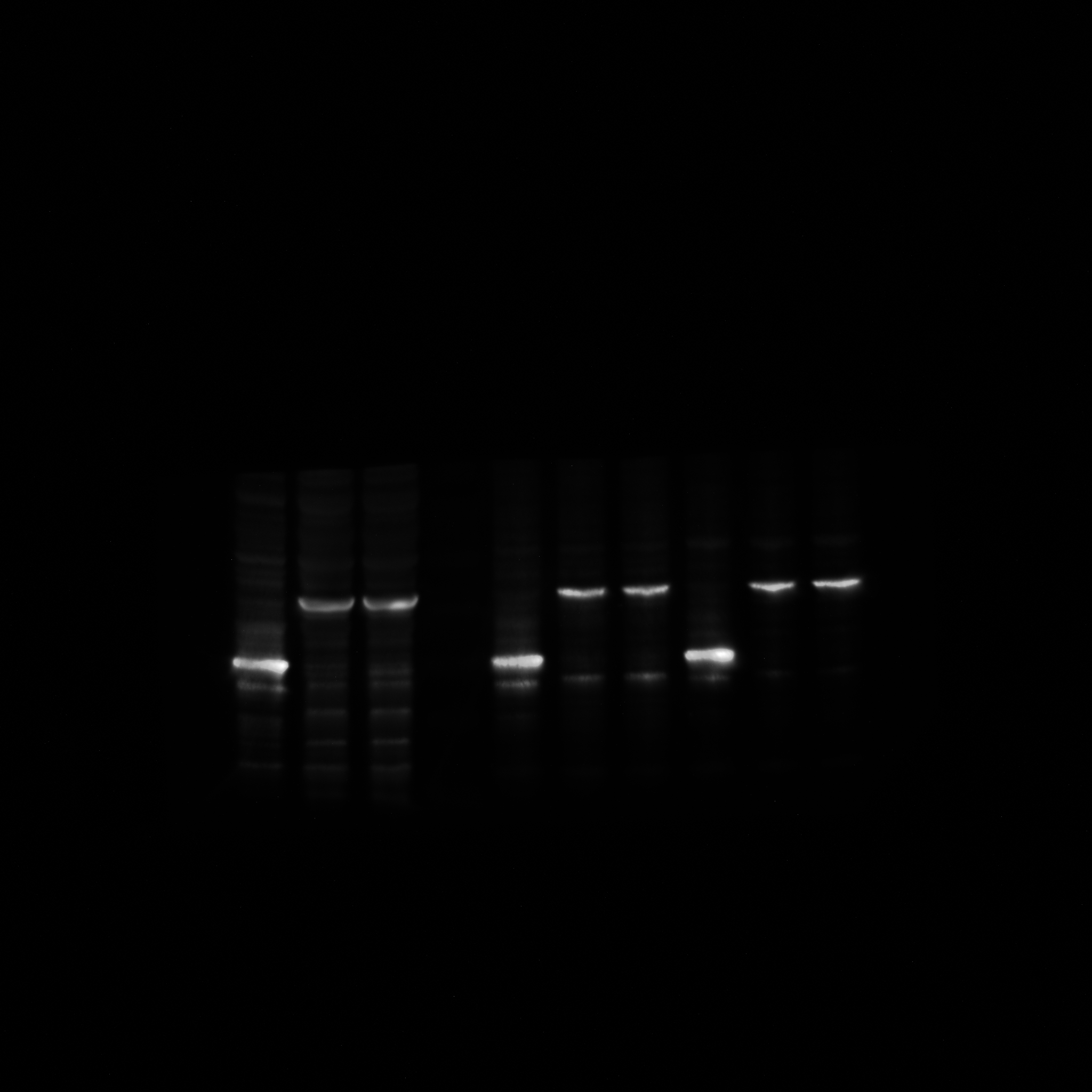

Supplement: Figure 1—source data 1. [file elife-84143-fig1-data1.zip › Fig_1D/EB1N_blot_1min_exposure.tif]

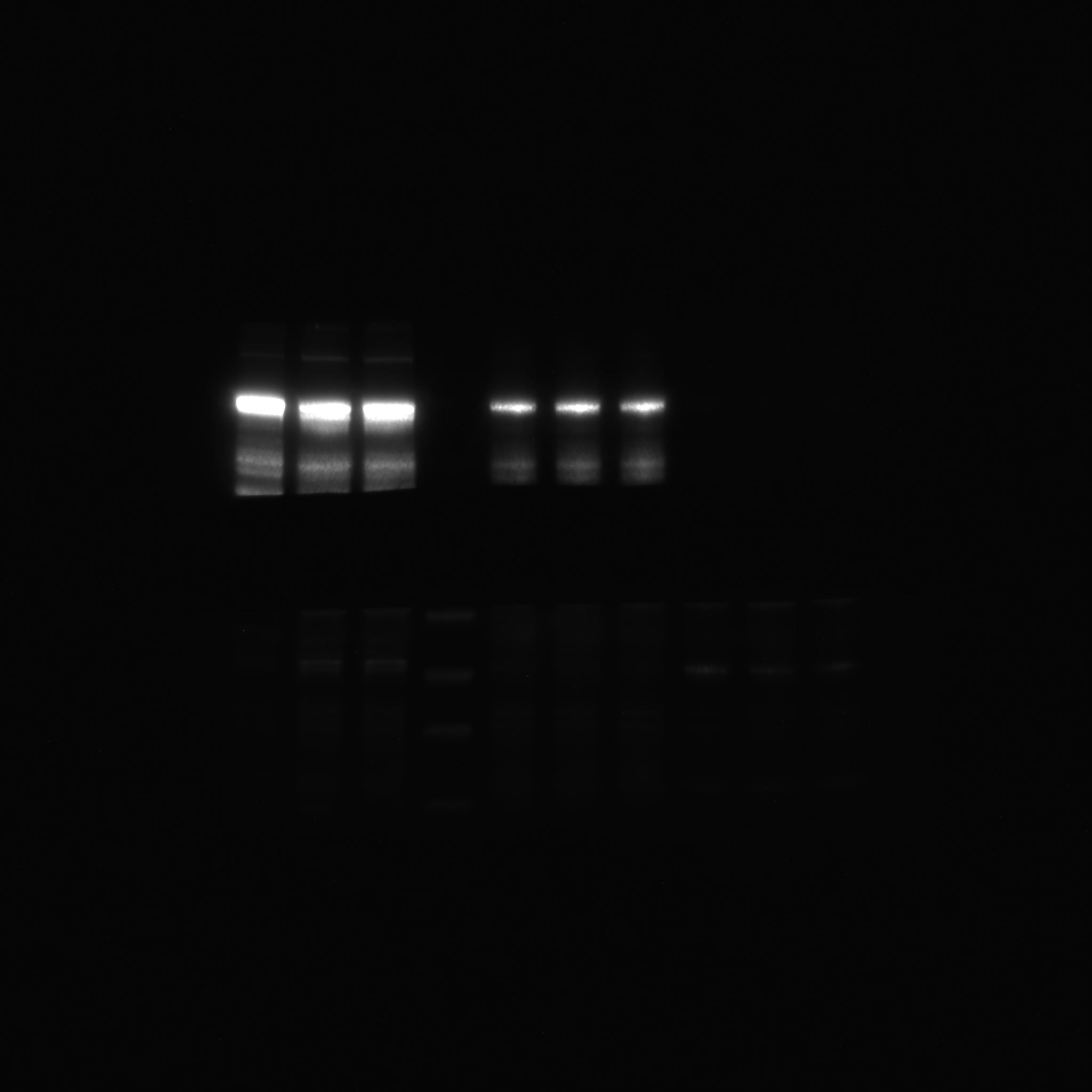

Supplement: Figure 1—source data 1. [file elife-84143-fig1-data1.zip › Fig_1D/EB3_blot(bottom)_20min_exposure.tif]

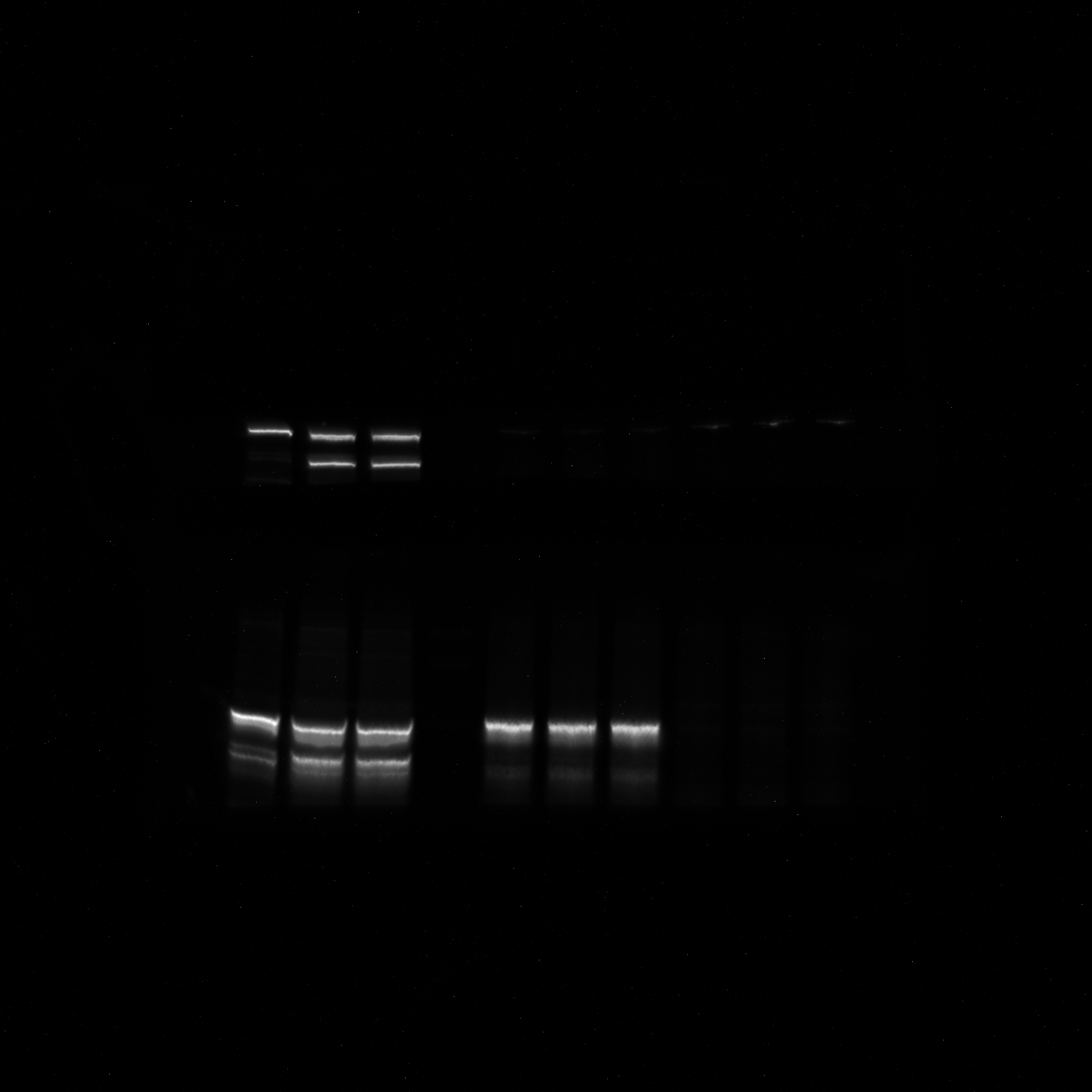

Supplement: Figure 1—source data 1. [file elife-84143-fig1-data1.zip › Fig_1D/KIF2C_blot(bottom)_5min_exposure.tif]

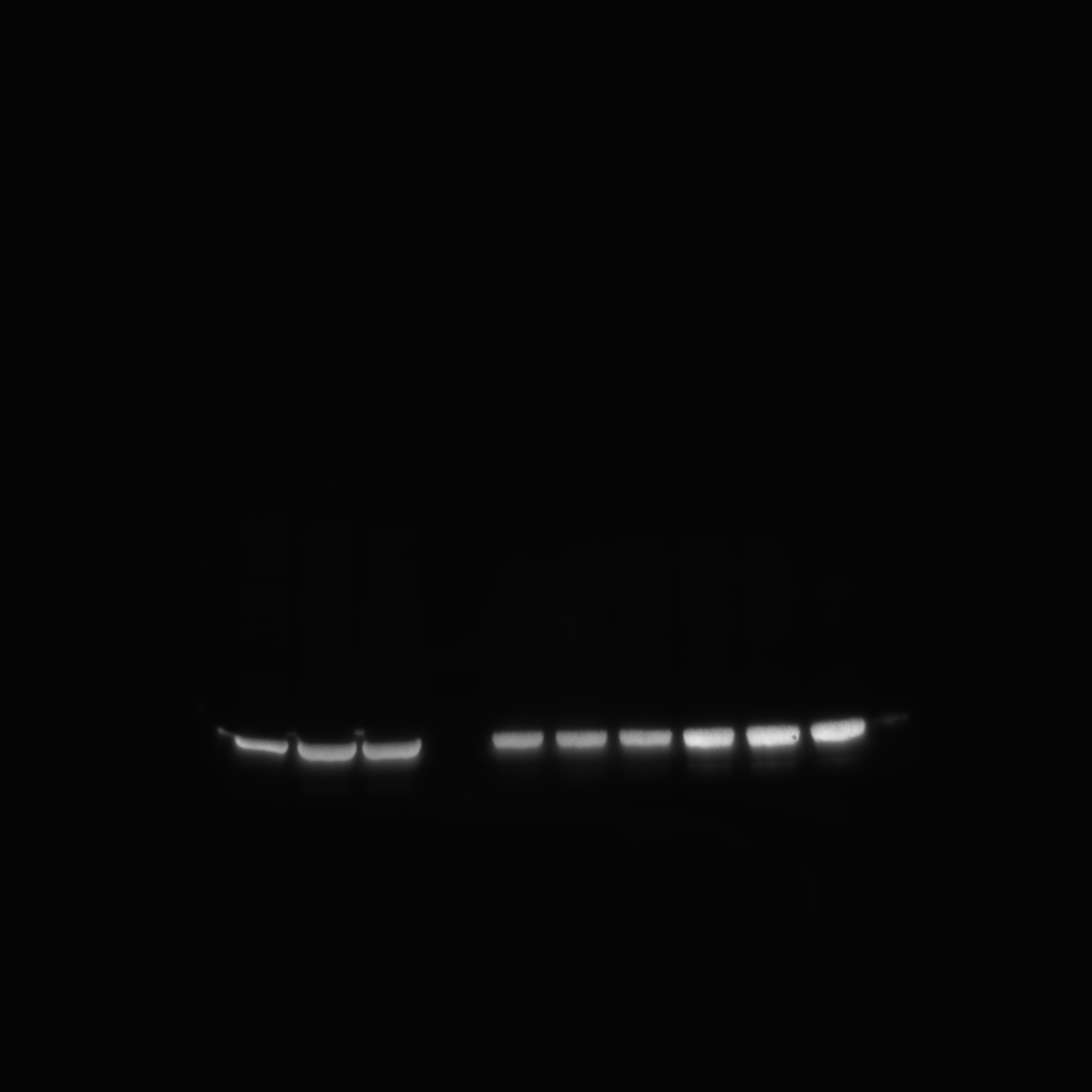

Supplement: Figure 1—source data 1. [file elife-84143-fig1-data1.zip › Fig_1D/tubulin_blot_40s_exposure.tif]

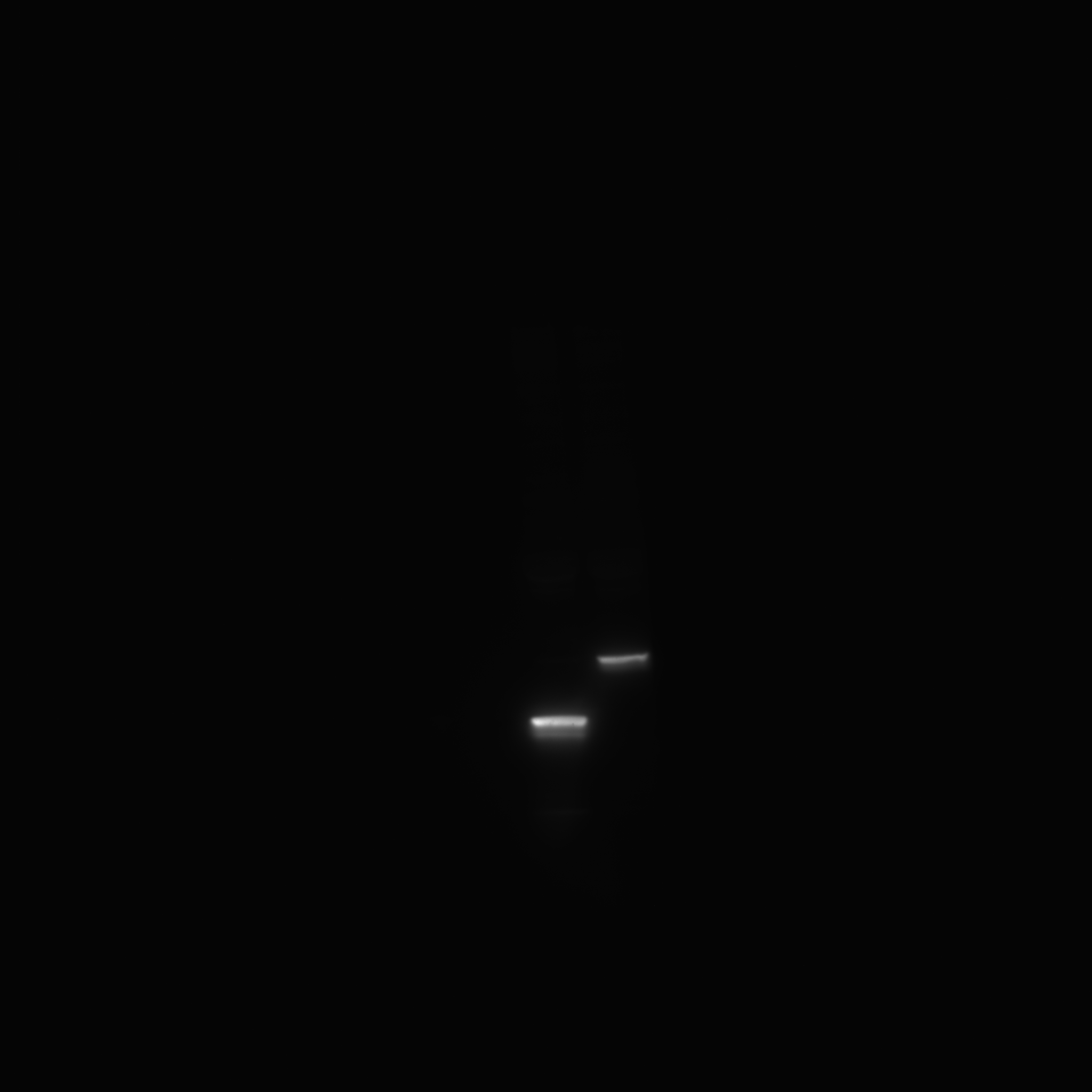

Supplement: Figure 1—source data 1. [file elife-84143-fig1-data1.zip › Fig_1H/EB1N_blot_10s_exposure.tif]

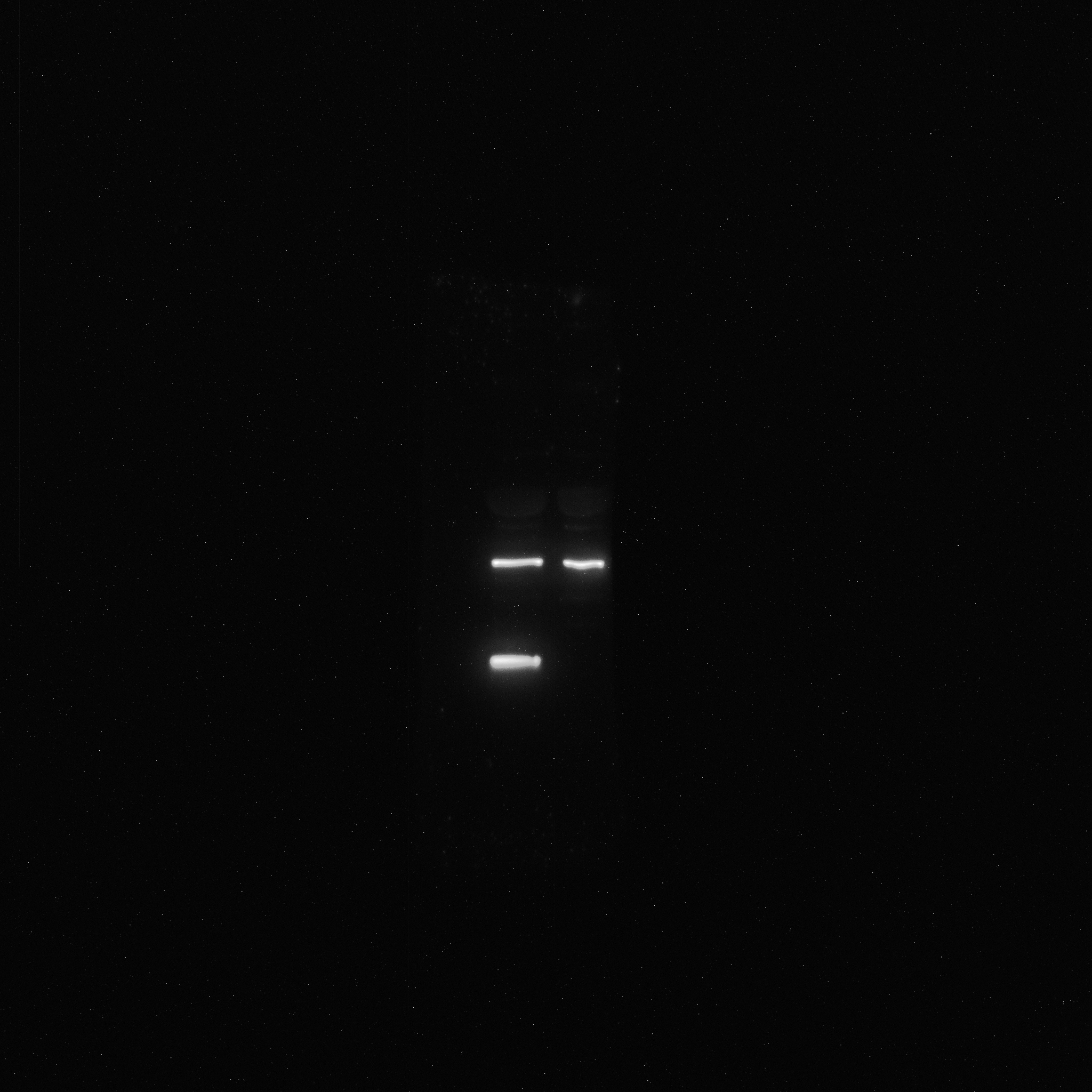

Supplement: Figure 1—source data 1. [file elife-84143-fig1-data1.zip › Fig_1H/EB3_blot_1min_exposure.tif]

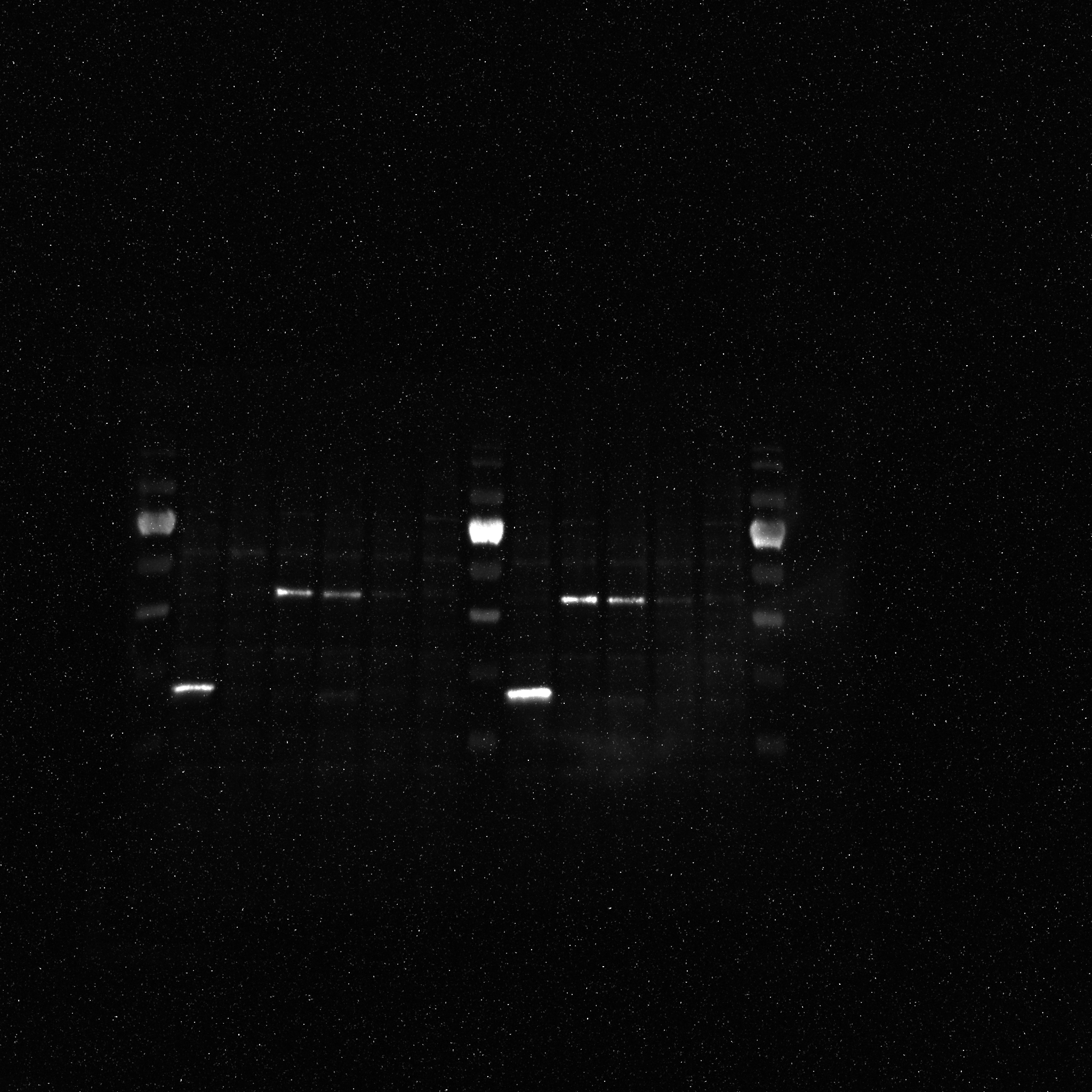

Supplement: Figure 1—figure supplement 1—source data 1. [file elife-84143-fig1-figsupp1-data1.zip › Fig_1_figure_supplement_1_source_data/EB1C_blot_20min_exposure.tif]

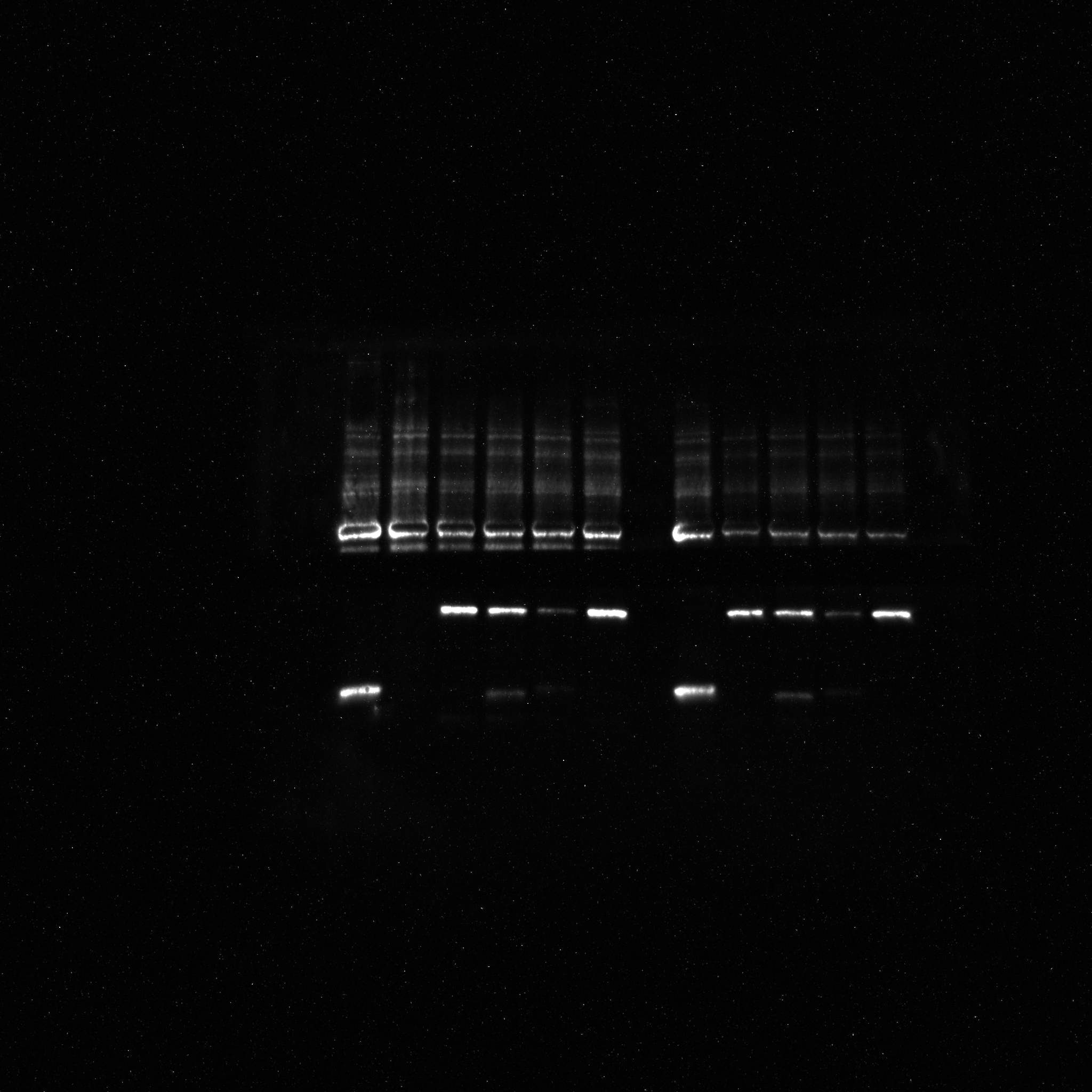

Supplement: Figure 1—figure supplement 1—source data 1. [file elife-84143-fig1-figsupp1-data1.zip › Fig_1_figure_supplement_1_source_data/EB1N_blot_10min_exposure.tif]

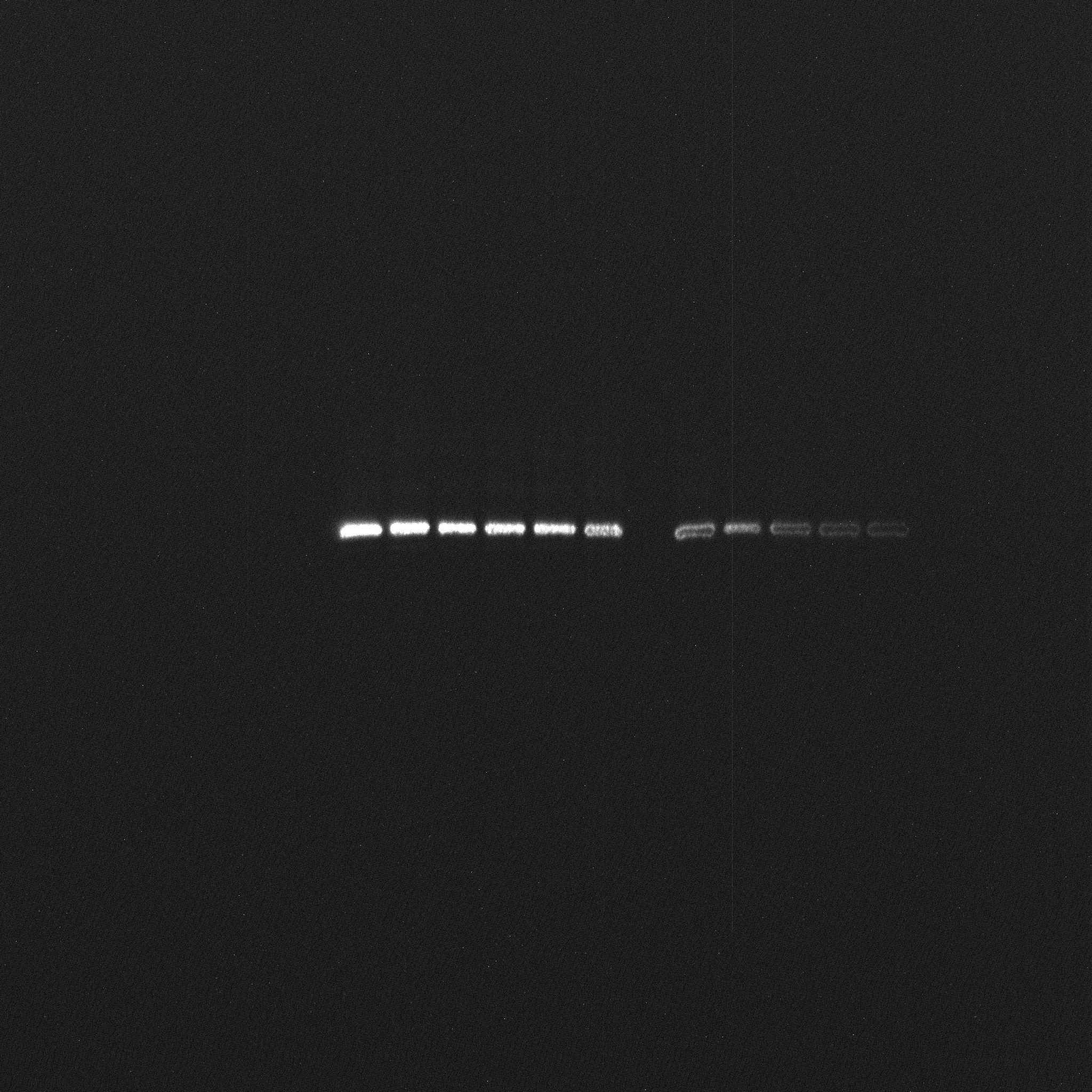

Supplement: Figure 1—figure supplement 1—source data 1. [file elife-84143-fig1-figsupp1-data1.zip › Fig_1_figure_supplement_1_source_data/tubulin_blot.tif]

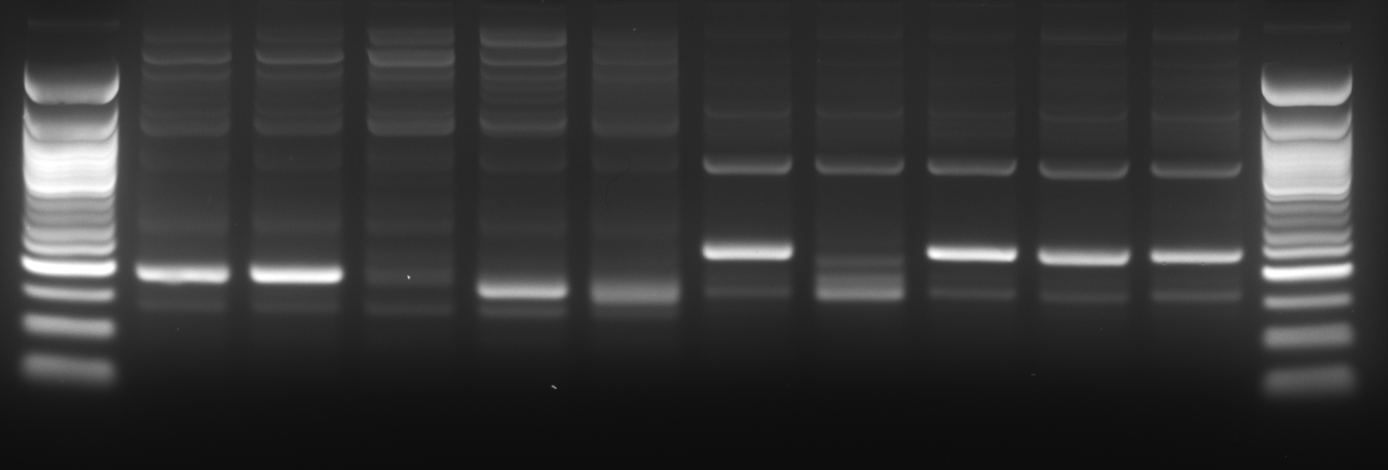

Supplement: Figure 1—figure supplement 1—source data 1. [file elife-84143-fig1-figsupp1-data1.zip › Fig_1_figure_supplement_1_source_data/pcr17_10_03.tif]

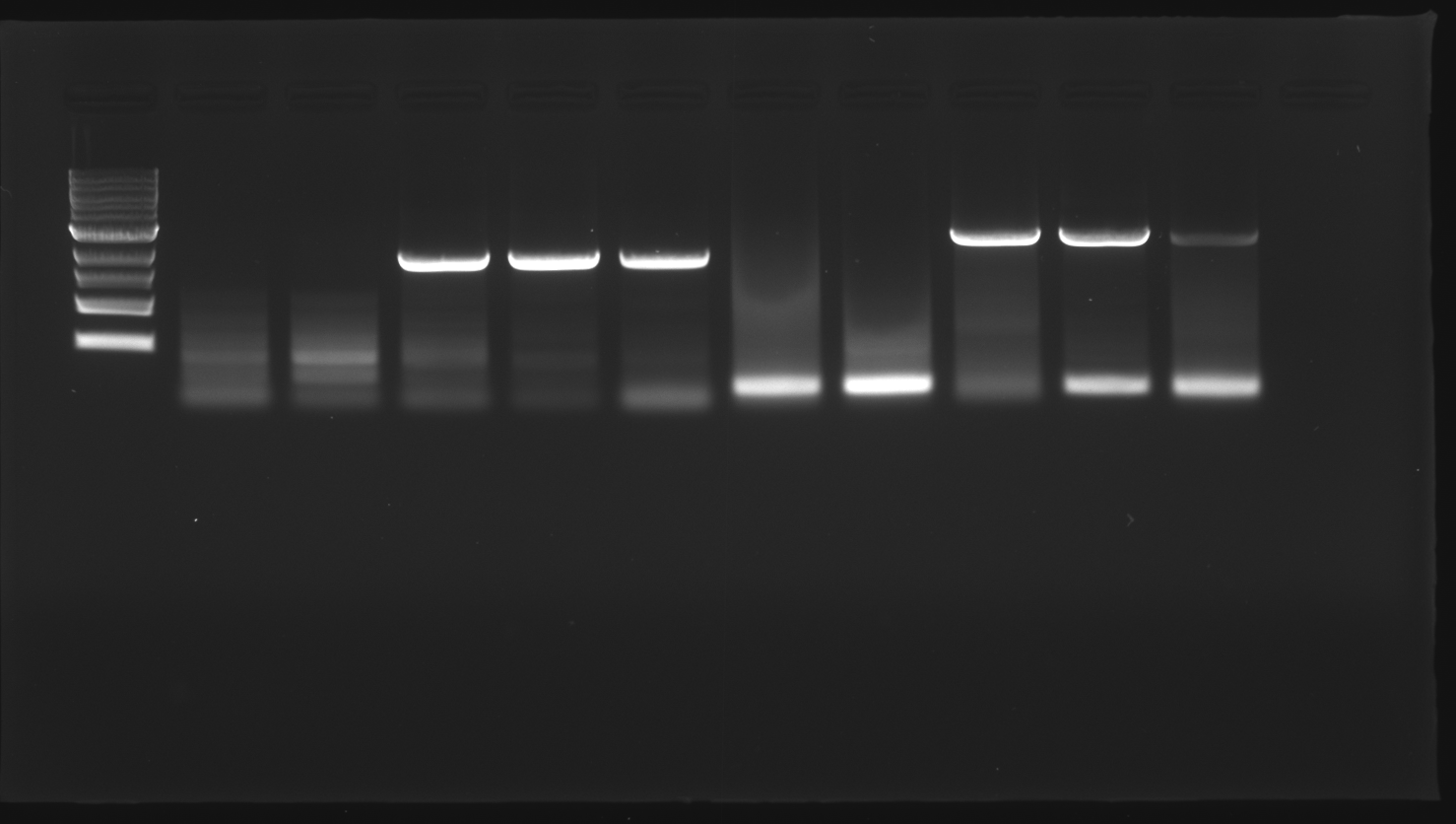

Supplement: Figure 1—figure supplement 1—source data 1. [file elife-84143-fig1-figsupp1-data1.zip › Fig_1_figure_supplement_1_source_data/pcr17_10_20.tif]

Figure 1 supplement 1 - source data

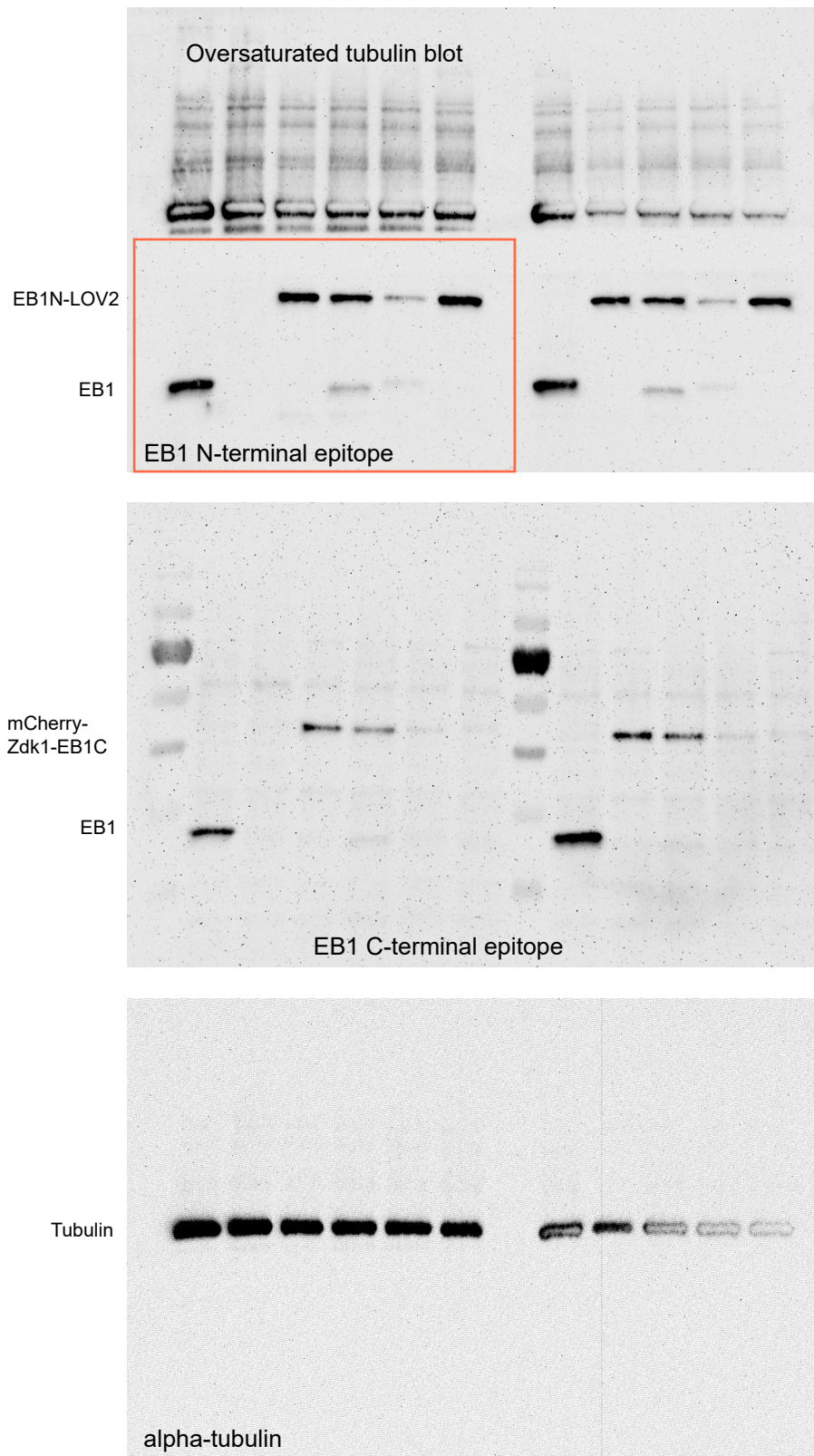

Figure 1 supplement 1 - source data

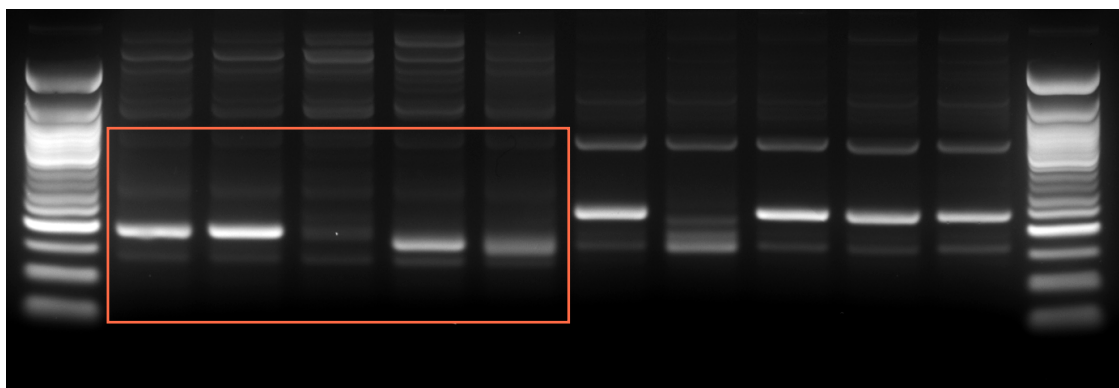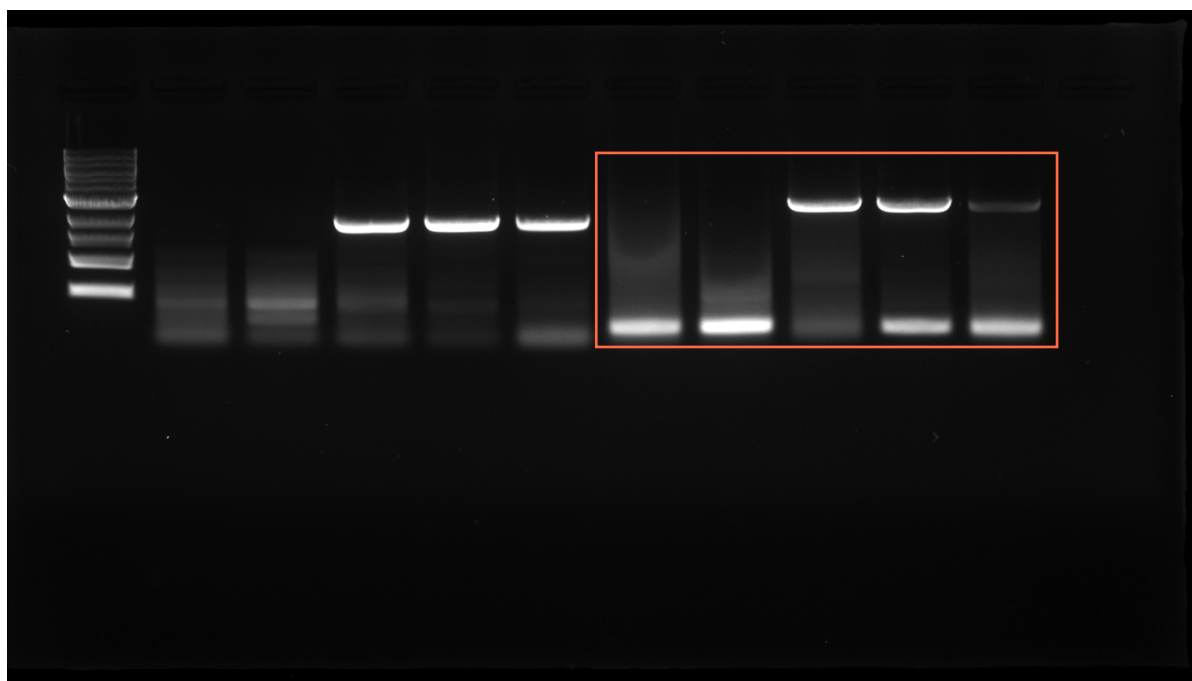

Supplement: Figure 1—figure supplement 1—source data 1. [file elife-84143-fig1-figsupp1-data1.zip › Fig_1_supplement_1_annotated_source_data.pdf]

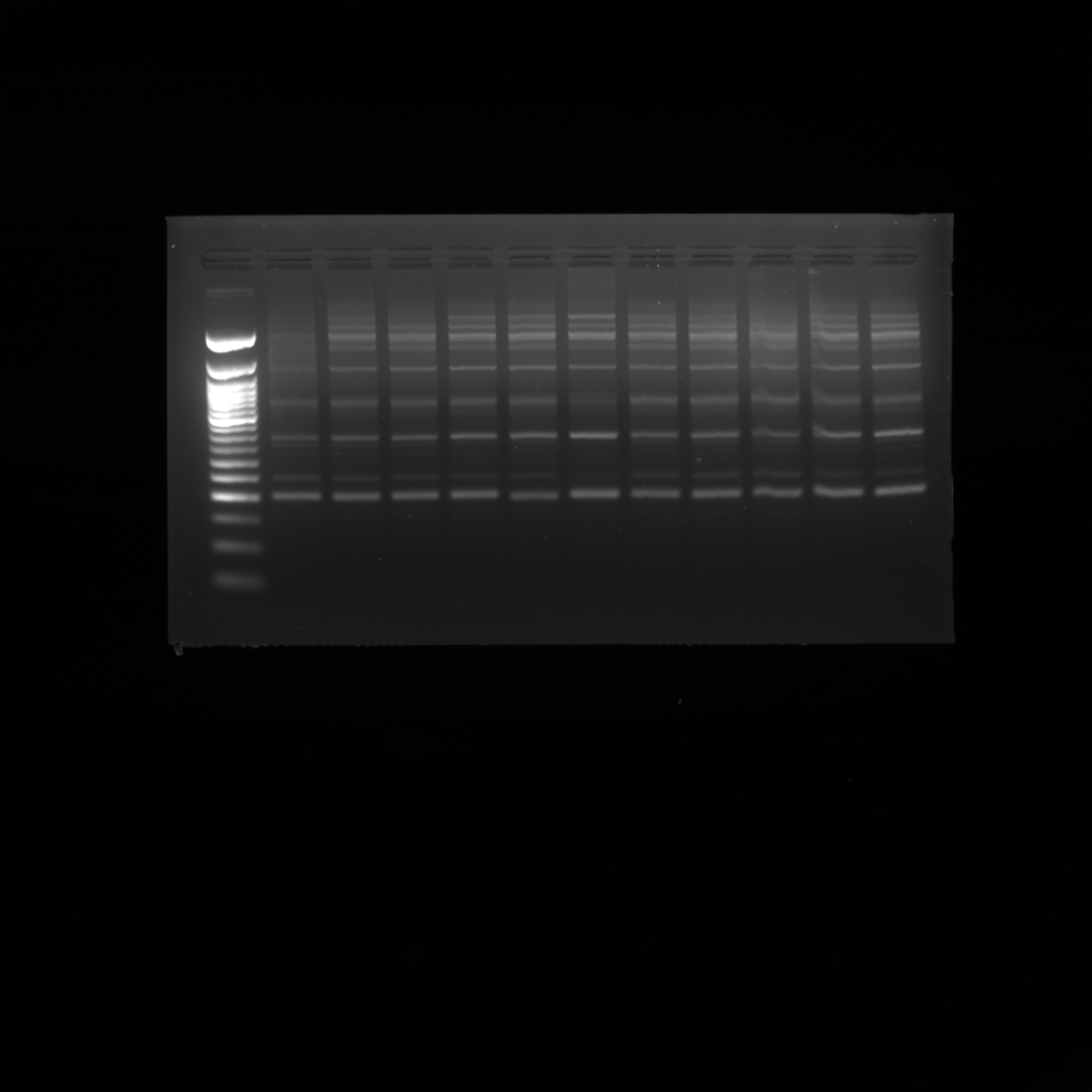

Supplement: Figure 1—figure supplement 3—source data 1. [file elife-84143-fig1-figsupp3-data1.zip › Fig_1_figure_supplement_3_source data/5-15 eb3 exon1 pcr2.tif]

**Figure 1 supplement 3 - source data**

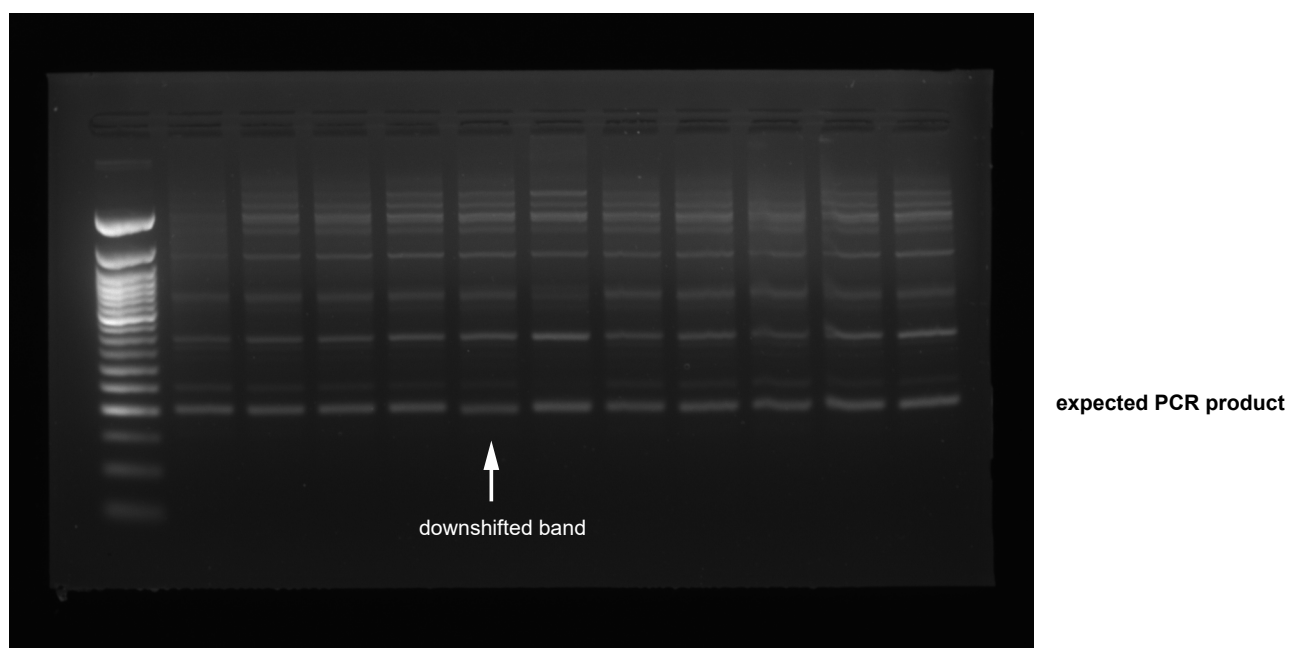

Supplement: Figure 1—figure supplement 3—source data 1. [file elife-84143-fig1-figsupp3-data1.zip › Fig_1_figure_supplement_3_annotated_source_data.pdf]
